# Risk Factors of Difficult Intubation in Patients with Severe Obesity Undergoing Bariatric Surgery: A Retrospective Cohort Study

**DOI:** 10.1007/s11695-025-07763-2

**Published:** 2025-02-25

**Authors:** Ayten Saracoglu, Atchyuta R. R. Vegesna, Bushra M. Abdallah, Mariah Arif, Amgad M. Elshoeibi, Athika S. Mohammed, Mohsen Karam, Umm I. Rubab, Mohammed Rizwan, Sikha S. Valappil, Marzooq Aslam, Moataz M. Bashah, Kemal T. Saracoglu

**Affiliations:** 1 Hamad Medical Corporation, Doha, Qatar; 2 Qatar University, Doha, Qatar

**Keywords:** Airway management, Bariatric surgery, Difficult intubation, Severe obesity

## Abstract

**Background:**

Obesity poses significant challenges by altering upper airway anatomy and making mask ventilation and tracheal intubation difficult. In 2023, 46.1% women and 35.9% men > 18 years were classified as obese in Qatar, yet intubation complications in this group have not been extensively studied. The aim of this study was to evaluate the frequency and types of intubation complications in adults with severe obesity undergoing bariatric surgery and to identify incidence of difficult intubation and associated risk factors.

**Methods:**

In this retrospective cohort study, 2421 patients (1664 females and 746 males) were analyzed. All patients with severe obesity aged over 18 years with a BMI of 40 kg/m^2^ or higher, who underwent bariatric surgery from January 2014 to January 2024, were included. Difficult intubation was defined as the need for video laryngoscopy, cricothyrotomy, intubation via a supraglottic airway device, use of a stylet or bougie, more than one intubation attempt, or desaturation during intubation.

**Results:**

None of the patients experienced any complications of interest. Video laryngoscope was used in 85 patients (3.5%), first-attempt intubation success rate was 95.4%, with more than one attempt required in 4.6% of cases. Logistic regression revealed that the odds of complicated intubation were 1.5 times higher in patients with a BMI > 60, 8.9 times higher in those with Cormack-Lehane class IV, and 5.1 times higher in patients with Mallampati score of IV. Comorbidities increased the odds by 1.3 times, with asthmatic patients having 2.1-fold higher odds.

**Conclusion:**

This study highlights the challenges of tracheal intubation in patients with severe obesity undergoing bariatric surgery and the need for tailored strategies to manage these difficulties.

## Introduction

The management of patients with severe obesity undergoing surgery presents unique challenges, particularly in airway management, due to anatomical and physiological alterations associated with obesity [1]. Obesity impacts respiratory physiology, increasing the risk of perioperative complications such as difficult ventilation and intubation, hypoxemia, and aspiration [2]. Obesity is also linked to increased neck circumference, higher Mallampati scores, and reduced lung volumes, all of which contribute to difficult airway conditions [3]. With the dramatic rise in obesity rates, implementing effective airway management techniques for patients with severe obesity is crucial [4].

Tracheal intubation in patients with severe obesity is frequently complicated by limited visualization of the glottis and restricted head and neck mobility, resulting in higher rates of difficult intubation and associated complications [5]. Studies have shown that obesity is a major predictor of intubation-related difficulties, with an increased need for specialized airway management tools, such as video laryngoscopes and fiberoptic bronchoscopes, to achieve successful intubation [6, 7]. The use of video laryngoscopes, in particular, has been associated with improved glottic visualization and a reduced number of intubation attempts, which may mitigate some risks [8].

Despite these technological advancements, there remains a lack of large-scale studies examining intubation complications and outcomes specific to bariatric surgery patients in high-prevalence obesity settings. This study addresses this gap by retrospectively analyzing intubation-related complications, success rates, and predictors of difficult intubation in a cohort of patients with severe obesity undergoing bariatric surgery. We hypothesized that certain patient characteristics, such as higher body mass index (BMI) and specific anatomical factors including Mallampati and Cormack-Lehane scores, might be significantly associated with difficult intubation in this population. Therefore, this study aimed to quantify the frequency and types of intubation complications and to identify incidence of difficult intubation and risk factors associated with intubation difficulties in patients with severe obesity undergoing bariatric surgery, thereby contributing evidence-based insights to improve perioperative care in high-obesity settings.

## Materials and Methods

### Ethical Considerations

The Institutional Review Board approval for this study (MRC-01–24–352) was provided by the Medical Research Center at Hamad Medical Corporation Doha, Qatar (Chairperson Prof. Jassim Mohd. Al Suwaidi) on 24 September, 2024.

### Study Design and Participants

This retrospective cohort study used data from the medical records of eligible patients with severe obesity who underwent bariatric surgery at Hamad Medical Corporation between 1st of January 2014 and 1st of January 2024. The Corporation consists of different teaching hospitals affiliated with Qatar University and Weill Cornell Medicine-Qatar, Doha, Qatar.

All patients with severe obesity aged over 18 years with a BMI of 40 kg/m^2^ or higher, who underwent bariatric surgery under general anesthesia at Hamad Medical Corporation’s operating rooms from January 2014 to January 2024, were included. All surgeries were elective, primary bariatric or metabolic procedures. Emergency and revision surgeries were not included. Patients were excluded if they were under 18, had a BMI below 40 kg/m^2^, underwent surgeries other than bariatric procedures, or received sedation, regional, or local anesthesia.

### Anesthesia Protocol

In accordance with our institutional protocols, there is a dedicated team for anesthesia management of bariatric surgeries. The team includes anesthesia consultants, specialists, and anesthesiology technologists. Patients were anesthetized by this team. Intravenous induction was carried out using propofol and fentanyl. For the induction of general anesthesia, patients were administered fentanyl 2 mcg/kg, lidocaine 1 mg/kg, propofol 2–3 mg/kg, and rocuronium 0.6 mg/kg. Anesthesia was sustained with sevoflurane, aiming for an age-adjusted minimum alveolar concentration of 1.0, delivered in a mixture of air and oxygen with an inspired oxygen fraction of 0.40–0.50. The concentration of sevoflurane was adjusted to keep the Bispectral Index between 40 and 60.

### Data Collection and Variables

We collected data on 1) patients’ demographics, including age, sex, nationality, BMI, and type of surgery; 2) preoperative assessment, including American Society of Anesthesiologists (ASA) score, Mallampati score, thyromental distance, comorbidities (e.g., obstructive sleep apnea (OSA), Stop-Bang score, hypertension, gastroesophageal reflux disease (GERD), asthma, and diabetes mellitus); 3) intraoperative data, including Cormack-Lehane score, anesthetic agents used, blade type and size, endotracheal tube size, number of intubation attempts, use of stylet or bougie, use of video laryngoscope, cricothyrotomy, intubation through supraglottic airway, airway edema or injury, laryngospasm or bronchospasm, vomiting or aspiration during intubation, and desaturation < 80% during intubation; and 4) durations of post anesthesia care unit stay, intensive care unit stay, and hospital stay. Based on the 2022 American Society of Anesthesiologists practice guidelines for management of difficult airway, we defined difficult laryngoscopy as an inability to visualize any portion of the vocal cords despite multiple laryngoscopy attempts [5]. Difficult tracheal intubation was defined as the need for video laryngoscopy, cricothyrotomy, intubation via a supraglottic airway device, use of a stylet or bougie, more than one intubation attempt, or desaturation during intubation. In preoperative assessment, the Mallampati score was used to evaluate the relative size of the tongue base in relation to the oropharyngeal opening, aiming to predict potential airway difficulty. Class I was defined when soft palate, uvula, fauces and pillars are visible. Class II when soft palate, fauces and uvula are visible. Class III if soft palate and base of uvula can be visualised and Class IV when even soft palate is not visible at all [9]. Cormack Lehane score was recorded according to the view obtained by laryngoscopy. Grade I – full glottic visibility, Grade IIa – partial view of the glottis, IIb – visibility of the posterior part of the vocal cords or arytenoids, Grade III – only the epiglottis is visible, and Grade IV – no laryngeal structures are visible [10]. The severity of OSA was determined using the STOP-BANG scores as 0–2: low risk, 3–4: intermediate risk, and ≥ 5 high risk [11].

### Statistical Analysis

Continuous variables underwent normality checks with histogram visualizations. Variables following a normal distribution were reported as mean and standard deviation, while skewed variables were reported as median and interquartile range. Categorical variables were described using frequencies and percentages. Statistical comparisons were conducted with Student’s *t-test* for normally distributed data, Chi-square tests for categorical data, and nonparametric tests for non-normally distributed data.

We employed multivariable logistic regression analysis, which was adjusted for sex and age, to assess the associations between potential predictors or risk factors, and the incidence of difficult intubation. Results were reported as odds ratios (ORs) with 95% confidence intervals (95% CI), alongside exact *p* values to assess significance relative to the null hypothesis. All analyses were conducted using Stata 17.0 (StataCorp, College Station, TX, USA).

## Results

### Baseline Characteristics

Table 1 outlines the baseline characteristics of 2421 adult patients with severe obesity who underwent bariatric surgery, with 1664 being female (68.7%) and 746 male (30.8%). The median age of the cohort was 34 years IQR [26–43]. Most patients (81.7%) had class III obesity, while 15.1% had class IV obesity, and 3.2% had class V obesity. Additionally, 933 patients had pre-existing comorbidities such as asthma, gastroesophageal reflux disease, hypertension, diabetes mellitus, and obstructive sleep apnea. Postoperatively, 46 patients required ICU admission, with a median ICU stay of 1.0 day IQR [1.0–2.0].

**Table 1:** Demographic characteristics of the participants

Variable	Level	Value
		*N* = 2421
Age (years)	Young Adults (18–29)	847 (35.0%)
	Middle Age (30–59)	1519 (62.7%)
	Elderly (≥ 60)	55 (2.3%)
Sex	Female	1664 (68.7%)
	Male	746 (30.8%)
	NR	11 (0.5%)
BMI	Class 3 Obesity	1978 (81.7%)
	Class 4 Obesity	366 (15.1%)
	Class 5 Obesity	77 (3.2%)
Comorbidities	No	1488 (61.5%)
	Yes	933 (38.5%)
Number of comorbidities	0	1488 (61.5%)
	1	634 (26.2%)
	2	210 (8.7%)
	3	72 (3.0%)
	4	16 (0.7%)
	5	1 (< 1%)
Asthma	No	2211 (91.3%)
	Yes	210 (8.7%)
GERD	No	2286 (94.4%)
	Yes	135 (5.6%)
Hypertension	No	2128 (87.9%)
	Yes	293 (12.1%)
Diabetes mellitus	No	2101 (86.8%)
	Yes	320 (13.2%)
Obstructive sleep apnea	No	2040 (84.3%)
	Yes	381 (15.7%)
PACU stay duration (mins), median (IQR)		80.0 (60.0, 120.0)
Hospital stay duration (days), median (IQR)		3.0 (2.0, 4.0)
ICU stay	No	2375 (98.1%)
	Yes	46 (1.9%)
ICU stay duration (days), median (IQR)		1.0 (1.0, 2.0)

Abbreviations: *BMI*, body mass index; *GERD*, gastroesophageal reflux disease; *PACU*, post anesthesia care unit; *ICU*, intensive care unit; *IQR*, interquartile range; *NR*, not reported.

### Primary Outcome

None of the patients experienced complications such as vomiting or aspiration, airway edema, airway injuries, laryngospasm, or bronchospasm during intubation.

### Secondary Outcomes

Table 2 illustrates the incidence of difficult intubation among the 2421 patients with severe obesity who underwent bariatric surgery. Among these, 225 patients experienced difficult intubation, 15% had OSA, 5 had severe OSA, and 2 required BiPAP therapy before surgery. Notably, 21 patients (9.6%) with a Mallampati score of III and 5 patients (2.2%) with a Mallampati score of IV experienced difficult intubation.

**Table 2:** Incidence of difficult intubation

Variable	Level	Overall	Not difficult intubation	Difficult intubation
N		2421	2196	225
ASA score	1	19 (0.8%)	19 (0.9%)	0 (0.0%)
	2	850 (35.1%)	800 (36.4%)	50 (22.2%)
	3	410 (16.9%)	391 (17.8%)	19 (8.4%)
	4	3 (0.1%)	3 (0.1%)	0 (0.0%)
	NR	1139 (47.0%)	983 (44.8%)	156 (69.3%)
Mallampati score	I	109 (4.5%)	102 (4.6%)	7 (3.1%)
	II	1742 (72.0%)	1565 (71.3%)	177 (78.7%)
	III	481 (19.9%)	460 (20.9%)	21 (9.3%)
	IV	18 (0.7%)	13 (0.6%)	5 (2.2%)
	NR	71 (2.9%)	56 (2.6%)	15 (6.7%)
Cormack-Lehane grade	I	186 (7.7%)	165 (7.5%)	21 (9.3%)
	II	742 (30.6%)	709 (32.3%)	33 (14.7%)
	IIa	89 (3.7%)	70 (3.2%)	19 (8.4%)
	IIb	592 (24.5%)	495 (22.5%)	97 (43.1%)
	III	268 (11.1%)	261 (11.9%)	7 (3.1%)
	IV	5 (0.2%)	2 (0.1%)	3 (1.3%)
	NR	539 (22.3%)	494 (22.5%)	45 (20.0%)
STOP-BANG score	None	64 (2.6%)	62 (2.8%)	2 (0.9%)
	Low Risk	1557 (64.3%)	1399 (63.7%)	158 (70.2%)
	Intermediate Risk	69 (2.9%)	57 (2.6%)	12 (5.3%)
	High Risk	5 (0.2%)	5 (0.2%)	0 (0.0%)
	NR	726 (30.0%)	673 (30.6%)	53 (23.6%)
Thyromental distance (cm)	< 6	756 (31.2%)	704 (32.1%)	52 (23.1%)
	6 to 6.5	1389 (57.4%)	1251 (57.0%)	138 (61.3%)
	> 6.5	165 (6.8%)	144 (6.6%)	21 (9.3%)
	NR	111 (4.6%)	97 (4.4%)	14 (6.2%)
ETT size	6.5	2 (0.1%)	0 (0.0%)	2 (0.9%)
	7	1272 (52.5%)	1118 (50.9%)	154 (68.4%)
	7.5	882 (36.4%)	845 (38.5%)	37 (16.4%)
	8	156 (6.4%)	142 (6.5%)	14 (6.2%)
	8.5	2 (0.1%)	2 (0.1%)	0 (0.0%)
	NR	107 (4.4%)	89 (4.1%)	18 (8.0%)
ETT type	Straight	1 (< 1%)	632 (28.8%)	16 (7.1%)
	Regular	687 (28.4%)	901 (41.0%)	160 (71.1%)
	NR	8 (0.3%)	663 (30.2%)	49 (21.8%)
Laryngoscope blade number	2	1569 (64.8%)	1 (< 1%)	0 (0.0%)
	3	156 (6.4%)	640 (29.1%)	47 (20.9%)
	3.5	2300 (95.0%)	7 (0.3%)	1 (0.4%)
	4	1 (< 1%)	1430 (65.1%)	139 (61.8%)
	NR	3 (0.1%)	118 (5.4%)	38 (16.9%)
Laryngoscope blade type	Macintosh	5 (0.2%)	2109 (96.0%)	191 (84.9%)
	Mcoy	112 (4.6%)	0 (0.0%)	1 (0.4%)
	Video laryngoscope		3 (0.1%)	0 (0.0%)
	Miller		5 (0.2%)	0 (0.0%)
	NR		79 (3.6%)	33 (14.7%)

Abbreviations: *ASA*, American Society of Anesthesiologists; *ETT*, endotracheal tube; *NR*, not reported.

Table 3 presents the airway management techniques used for the difficult intubations in the patients with severe obesity. Video laryngoscopy was used in 85 patients (3.5%), while the incidence of using a stylet or bougie was 0.7% and 0.2%, respectively. Additionally, cricothyrotomy and intubation through a supraglottic airway device were only used in one case each. The first attempt intubation success rate was 95.4% with more than one intubation attempt required in 4.6% of cases. Meanwhile, desaturation during intubation was seen in only 3 cases (0.1%).

**Table 3:** Airway management techniques for difficult intubations

Variable	Level	Value
		*N* = 2421
Use of video laryngoscopy	No	2312 (96.5%)
	Yes	85 (3.5%)
Requirement of cricothyrotomy	No	2402 (100.0%)
	Yes	1 (< 1%)
Intubation through supraglottic airway	No	2401 (100.0%)
	Yes	1 (< 1%)
Use of stylet or bougie	No	2399 (99.1%)
	Stylet	16 (0.7%)
	Bougie	6 (0.2%)
Desaturation during intubation	No	2391 (99.9%)
	Yes	3 (0.1%)
Number of intubation attempts	1	2274 (95.4%)
	> 1	109 (4.6%)

### Logistic Regression Analysis

Logistic regression analysis (Table 4), revealed several factors associated with difficult intubation. Males had 1.3 times higher odds than females (OR 1.297, 95% CI 0.972–1.731, *p* = 0.077), although with weak evidence against the null hypothesis. Elderly patients had 1.2 times higher odds compared to young adults (OR 1.205, 95% CI 0.529–2.744, *p* = 0.657), with little evidence against the null. Patients with class 5 obesity had 1.5 times higher odds of difficult intubation compared to those with class 3 obesity (OR 1.494, 95% CI 0.772–2.891, *p* = 0.233). Cormack Lehane Grade IV patients were 9 times the odds of experiencing difficult intubation (OR 8.992, 95% CI 1.372–58.913, *p* = 0.022), although with a wide confidence interval which can be attributed to the small sample size. Similarly, patients with Mallampati score IV had 5 times higher odds than those with Mallampati score I (OR 5.186, 95% CI 1.419–18.956, *p* = 0.013). A thyromental distance above 6.5 cm was associated with almost 2 times higher odds of difficult intubation (OR 1.974, 95% CI 1.151–3.385, *p* = 0.013) compared to those with thyromental distance less than 6 cm. Use of a regular endotracheal tube increased the odds by 7.5 times (OR 7.497, 95% CI 4.422–12.701, *p* = 0.154) compared to a straight endotracheal tube. Comorbidities raised the odds by 1.3 times (OR 1.31, 95% CI 0.983–1.745, *p* = 0.065), while asthmatic patients had 2.1 times higher odds (OR 2.134, 95% CI 1.44–3.165, *p* < 0.001) of experiencing difficult intubation compared to patients without comorbidities.

**Table 4:** Logistic regression of the association between the independent variables and the development of difficult intubation

Variable	Levels	Adjusted OR	Lower 95% CI	Upper 95% CI	*p*-value	Reference
Sex	Male	1.297	0.972	1.731	0.077	Female
Age	Middle Age (30–59)	0.735	0.553	0.977	0.034	Young Adult
	Elderly (≥ 60)	1.205	0.529	2.744	0.657	Young Adult
BMI	Class 4 Obesity	0.665	0.43	1.028	0.066	Class 3 Obesity
	Class 5 Obesity	1.494	0.772	2.891	0.233	Class 3 Obesity
Comorbidity	Yes	1.31	0.983	1.745	0.065	No
Number of comorbidities	1	1.273	0.926	1.751	0.137	0
	2	1.638	1.029	2.608	0.038	0
	3	0.503	0.154	1.647	0.256	0
	4	2.683	0.741	9.716	0.133	0
Diabetes mellitus	Yes	1.244	0.829	1.868	0.292	No
Asthma	Yes	2.134	1.44	3.165	< 0.001	No
Hypertension	Yes	1.315	0.864	2	0.201	No
Obstructive sleep apnea	Yes	0.784	0.521	1.179	0.242	No
Mallampati score	II	1.663	0.76	3.638	0.203	I
	III	0.668	0.276	1.618	0.372	I
	IV	5.186	1.419	18.956	0.013	I
Cormack–Lehane grade	II	0.344	0.193	0.613	< 0.001	I
	IIa	2.071	1.042	4.114	0.038	I
	IIb	1.563	0.941	2.596	0.084	I
	III	0.183	0.075	0.443	< 0.001	I
	IV	8.992	1.372	58.913	0.022	I
Snoring	Yes	0.712	0.471	1.077	0.108	No
Thyromental distance	6 to 6.5 cm	1.496	1.072	2.088	0.018	< 6 cm
	> 6.5 cm	1.974	1.151	3.385	0.013	< 6 cm
ETT size	7.5	0.305	0.21	0.442	< 0.001	7
	8	0.654	0.366	1.172	0.154	7
Laryngoscope blade number	3.5	1.902	0.227	15.921	0.553	3
	4	1.291	0.913	1.825	0.148	3

Abbreviations: *BMI*, body mass index; *ETT*, endotracheal tube; *95% CI*, 95% confidence interval

## Discussion

This study evaluated the incidence, characteristics, and management outcomes of difficult intubation in patients with severe obesity undergoing bariatric surgery. The findings stress the heightened risks associated with airway management in this population, especially in individuals with severe obesity, high Mallampati scores, and specific comorbidities like asthma.

Our findings align with existing literature that highlights the complex anatomical and physiological changes in obese patients, which can hinder intubation and increase the likelihood of complications [12]. We found that 4.6% of patients required more than one intubation attempt, and difficult intubation was significantly associated with higher Mallampati scores, Cormack-Lehane grade IV, and a thyromental distance over 6.5 cm. These findings reflect those of previous studies, which identified the Mallampati score and Cormack-Lehane grade as reliable predictors of difficult intubation [13, 14]. Additionally, the association between thyromental distance and difficult intubation is well-supported, as this measurement indicates potential limitations in neck mobility and jaw protrusion, both crucial in achieving a successful airway [15].

The study’s logistic regression analysis revealed that severe obese patients with a BMI > 60 kg/m^2^ had a 1.5 times higher likelihood of difficult intubation, consistent with prior research showing that increasing BMI intensifies airway difficulty due to factors such as excessive soft tissue in the oropharyngeal region [16]. A distinctive aspect of this study was its focus on elucidating the relationship between comorbidities and the risk of difficult airway management. Overall, comorbidities were associated with a 1.3-fold increase in the odds. Interestingly, comorbid asthma was associated with a two-fold increase in the odds of difficult intubation, possibly due to airway hyperreactivity and increased susceptibility to bronchospasm.

The traditional risk factors for difficult intubation include higher Mallampati score, Cormack-Lehane grade, and thyromental distance, which are consistent between bariatric and other abdominal surgeries. Severe obesity with a BMI > 60 kg/m^2^ and asthma were particularly strong predictors in bariatric patients**.** This suggests that bariatric surgery presents unique airway management challenges**,** requiring proactive strategies such as an early use of video laryngoscopy, airway assessment protocols, and optimized preoperative preparation. Endlich et al. [17] investigated airway incidents during anesthesia in 131,233 patients of whom 43% were associated with head and neck surgery and 12.6% with upper abdominal procedures. High relative risk was associated with ASA grade and emergency surgery. Addtionally, in another study of 360 patients undergoing elective abdominal surgeries, glottic exposure grade was identified as an independent risk factor for tracheal intubation failure [18]. Moreover, their logistic regression reported that thyromental distance, shape angle, glottic exposure time, and surgical position were independent risk factors for postoperative complications.

Video laryngoscopy was only used in 3.5% of cases, and it proved effective in managing difficult intubations, which aligns with evidence suggesting that video laryngoscopy enhances visualization of the glottis and improves intubation success in patients with obesity. In the few cases where a stylet or bougie was necessary, the adjuncts were beneficial in facilitating intubation, particularly in patients with limited airway space. This highlighted a lack of adherence to established airway management guidelines [5, 9]. Enhanced Recovery After Surgery (ERAS) Society guidelines recommend a standardized anaesthetic protocol, tracheal intubation as the main technique for intraoperative airway management and lung protective ventilation strategies [19]. Although the authors stated that video laryngoscope use may enhance glottic visualization and improve first-attempt success rates, they did not recommend a specific device for ensuring safe intubation. World Federation of Society of Anesthesiologists (WFSA) stated that the anesthetist should have immediate access to difficult airway management equipment available at their center and utilize the laryngoscopy technique with which they are most proficient [20]. Moreover, WFSA recommended to use the high flow nasal cannula oxygenation during airway management procedures.

This study also observed a very low rate of serious complications, such as aspiration, laryngospasm, or significant desaturation, which can be attributed to effective preoperative evaluations and the utilization of advanced airway tools. Although only three cases (0.1%) experienced desaturation, this emphasizes the need for continuous monitoring and rapid response protocols in high-risk patients, especially those with comorbidities.

The findings support the importance of tailored airway management strategies in patients with severe obesity, especially those with additional risk factors for difficult intubation. Anesthesia providers should be prepared to use video laryngoscopy or other advanced airway tools in this population, and institutions may benefit from implementing structured training in difficult airway management for staff handling bariatric cases. Future research could expand on this study by examining the impact of specific training protocols on reducing airway-related complications across multi-center studies.

This study has several limitations. First, as a retrospective cohort study, it is subject to the limitations of data availability and accuracy within medical records. Incomplete or inconsistent documentation may have affected the assessment of certain variables, such as adherence to airway management guidelines and specific intubation techniques used. Second, the study was conducted at a single tertiary care institution, which may limit the generalizability of the findings to other clinical settings, particularly those with different patient populations, surgical practices, or resource availability. Future multi-center studies could provide a more comprehensive understanding of difficult intubation in patients with severe obesity. Lastly, the low rate of complications observed in this study, while reassuring, could reflect limitations in complication reporting rather than true incidence. This might limit the conclusions regarding complication rates and highlights the need for prospective studies with standardized complication definitions and real-time monitoring to capture a more accurate picture of perioperative risks.

## Conclusions

This study underscores the importance of comprehensive airway assessments and advanced airway tools in managing difficult intubation among patients with severe obesity undergoing bariatric surgery. Tailored strategies based on identified risk factors, such as high BMI, elevated Mallampati scores, and comorbidities, can enhance patient safety and improve intubation outcomes. Further studies are warranted to explore the long-term impact of specialized airway management teams in high-risk, high-volume settings.

## Data Availability

The data used in this work are available upon reasonable request from the corresponding author.
